# Synchronous Changes of Cortical Thickness and Corresponding White Matter Microstructure During Brain Development Accessed by Diffusion MRI Tractography from Parcellated Cortex

**DOI:** 10.3389/fnana.2015.00158

**Published:** 2015-12-02

**Authors:** Tina Jeon, Virendra Mishra, Minhui Ouyang, Min Chen, Hao Huang

**Affiliations:** ^1^Radiology Research, Children’s Hospital of Philadelphia, PhiladelphiaPA, USA; ^2^Advanced Imaging Research Center, University of Texas Southwestern Medical Center at Dallas, DallasTX, USA; ^3^Lou Ruvo Center for Brain Health, Cleveland Clinic, Las VegasNV, USA; ^4^Department of Mathematical Sciences, University of Texas at Dallas, RichardsonTX, USA; ^5^Department of Radiology, Perelman School of Medicine, University of Pennsylvania, PhiladelphiaPA, USA

**Keywords:** brain development, white matter microstructure, circuits, tractography, cortical thickness, synchronous

## Abstract

Cortical thickness (CT) changes during normal brain development is associated with complicated cellular and molecular processes including synaptic pruning and apoptosis. In parallel, the microstructural enhancement of developmental white matter (WM) axons with their neuronal bodies in the cerebral cortex has been widely reported with measurements of metrics derived from diffusion tensor imaging (DTI), especially fractional anisotropy (FA). We hypothesized that the changes of CT and microstructural enhancement of corresponding axons are highly interacted during development. DTI and T1-weighted images of 50 healthy children and adolescents between the ages of 7 and 25 years were acquired. With the parcellated cortical gyri transformed from T1-weighted images to DTI space as the tractography seeds, probabilistic tracking was performed to delineate the WM fibers traced from specific parcellated cortical regions. CT was measured at certain cortical regions and FA was measured from the WM fibers traced from same cortical regions. The CT of all frontal cortical gyri, including Brodmann areas 4, 6, 8, 9, 10, 11, 44, 45, 46, and 47, decreased significantly and heterogeneously; concurrently, significant, and heterogeneous increases of FA of WM traced from corresponding regions were found. We further revealed significant correlation between the slopes of the CT decrease and the slopes of corresponding WM FA increase in all frontal cortical gyri, suggesting coherent cortical pruning and corresponding WM microstructural enhancement. Such correlation was not found in cortical regions other than frontal cortex. The molecular and cellular mechanisms of these synchronous changes may be associated with overlapping signaling pathways of axonal guidance, synaptic pruning, neuronal apoptosis, and more prevalent interstitial neurons in the prefrontal cortex. Revealing the coherence of cortical and WM structural changes during development may open a new window for understanding the underlying mechanisms of developing brain circuits and structural abnormality associated with mental disorders.

## Introduction

The cerebral cortex contains neuronal bodies, to which afferent and efferent axonal fibers are connected. Perpendicular to the cortical surface, the cerebral cortex contains six layers. Despite substantial overlap, layer III is the major source of corticocortical fibers, layer V of corticostriate fibers, fibers to the brainstem, spinal cord, and layer VI of corticothalamic fibers (Nolte, 2002). Human brain development is remarkably complicated yet organized. During development, arborization takes place in the cerebral cortex and nerve fibers branch out to form brain circuits. Peak cortical thickness (CT) for some areas such as somatic sensory cortex is reached around 7 years (Shaw et al., 2008). Then, the branching stops and the brain starts to prune away some connections of the cerebral cortex (Giedd, 2004). As a result, cerebral CT undergoes an increase at the beginning years and decreases after around 7–10 years. Cortical thinning during brain development is related to complicated cellular and molecular processes including increased proliferation of myelin into the cortical neurophil, synaptic pruning, trophic glial, and vascular changes, cell shrinkage (Gogtay et al., 2004; Shaw et al., 2008) and apoptosis (Cowan, 1973; Morrison and Hof, 1997; Vanderhaeghen and Cheng, 2010). Although why the brain goes through neural pruning is not yet fully understood, one hypothesis is that the brain prunes away unused connections (Giedd, 2004). In addition, pruning of the cerebral cortex occurs in an asynchronous manner. A previous study (Shaw et al., 2008) has shown that cortical maturation rates and CT trajectories from childhood to adulthood vary regionally.

Heterogeneous developmental patterns have also been found for white matter (WM) axons. Most cerebral WM axons are those projected from neurons in the cerebral cortex. WM axons undergo dramatic changes during development, including axonal packing and myelination (Yakovlev and Lecours, 1967; Stiles and Jernigan, 2010). Electrical impulses across axons contribute to myelination, suggesting that training and experience during brain development may enhance myelination (Demerens et al., 1996). Differentiated WM axonal developmental processes were found from the fetal stage to early childhood (Huang et al., 2006, 2009; Dubois et al., 2008) and from childhood to adulthood (e.g., Lebel and Beaulieu, 2011). Taken together, previous findings suggest that the CT reduction and corresponding WM microstructural enhancement during brain development are intrinsically related. However, the relationship of these two processes is poorly understood. These parallel processes are essentially related to the formation of the brain circuits and mental disorders. For example, it has been suggested that excessive pruning and insufficient pruning are related to schizophrenia (Keshavan et al., 1994) and autism (Barnea-Goraly et al., 2004), respectively. It has also been shown that schizophrenia is linked to abnormalities of both WM microstructure and CT (Ehrlich et al., 2014). Therefore, the overarching hypothesis of this study is that changes of CT and WM microstructure are not independent, but highly interacted during brain development for the formation of brain circuits.

Advances in MR imaging techniques have made non-invasive and concurrent measurements of CT and WM axonal microstructure possible with T1-weighted imaging and diffusion MRI (dMRI) acquired in the same session. TI-weighted MRI offers high contrast of the cerebral cortex and can be used for CT measurements (e.g., Fischl and Dale, 2000). Early CT increases followed by decreases from around 7–10 years of age in development have been well documented with T1-weighted MRI (e.g., Giedd, 2004; Gogtay et al., 2004). Diffusion tensor imaging (DTI; Basser et al., 1994), using a tensor model based on dMRI dataset, is a type of MRI technique that measures the water diffusion properties in brain tissue. DTI-derived metrics are sensitive to microstructural changes of brain WM axons. Fractional anisotropy (FA; Moseley et al., 1990; Pierpaoli et al., 1996; Beaulieu, 2002), derived from the diffusion tensor, characterizes the degree of anisotropy of diffusion and has been widely used to quantify WM axonal microstructure. It has been found that WM FA, which is sensitive to axonal microstructural enhancement, increases dramatically during development (e.g., Beaulieu, 2002; Lebel et al., 2008; Westlye et al., 2010; Lebel and Beaulieu, 2011).

Coherence of changes of CT and FA of WM fibers traced from corresponding cortical regions during brain development may be related to structural connectivity. The structural connectivity can be characterized in two different ways, one with dMRI and the other with structural MRI (i.e., T1 or T2 weighted images). With dMRI tractography (e.g., Mori et al., 1999; Behrens et al., 2007), WM fibers can be traced to infer structural connectivity. With structural MRI, two brain regions are also considered anatomically connected if statistically significant correlations were found for CT at these two regions (He et al., 2007). Based on WM tractography with dMRI, numerous studies have been conducted in the past several years to characterize brain connectivity from a macroscopic perspective by application of graph theory analysis (Bullmore and Sporns, 2009). For example, increased efficiency and connectivity strength (e.g., Hagmann et al., 2010; Huang et al., 2015) based on tractography with dMRI have been found during brain development. Despite these macroscopic findings from graph theory analysis, the relationship of these two parameters (CT and corresponding WM FA) that can be used for constructing brain structural networks remains unknown. Most brain WM consists of axons projected from the neurons in the cerebral cortex. Neurons and associated WM axons behave coherently in development (Budhachandra et al., 2012). The relationship of CT and WM microstructure of developing brains has been investigated recently (Tamnes et al., 2010a; Wu et al., 2014; Smyser et al., 2015) with a different approach from that used in the present study. Specifically, the relationship between cortical regions and the adjacent WM, namely WM confined by certain physical distances from a certain cortical region, were focused on in those studies. To our knowledge, no investigation has been conducted to test the coherence between regional age-dependent CT decreases and age-dependent FA increases of the WM fibers traced from the corresponding cortical region, regardless of physical distance between the cortical region and traced WM fibers. We hypothesize that CT decreases of certain cortical regions and FA increases of WM fibers traced from the same cortical region are coherent. Revealing the link of the age-dependent changes of CT and those of corresponding WM FA could offer a refreshing perspective to delineate the intrinsic coherence of cortical regions and their connectional pathways. In this study, DTI and T1-weighted images were acquired from 50 normal children and adolescents at 7–25 years of age. WM fibers were directly traced from a certain parcellated cortical gyrus. The age-dependent CT decreases at certain cortical gyri, age-dependent increases of FA of WM fibers traced from corresponding cortical gyri, and the relationship of the changes of CT and those of FA were investigated.

## Materials and Methods

### Participants

**FIGURE 1:**
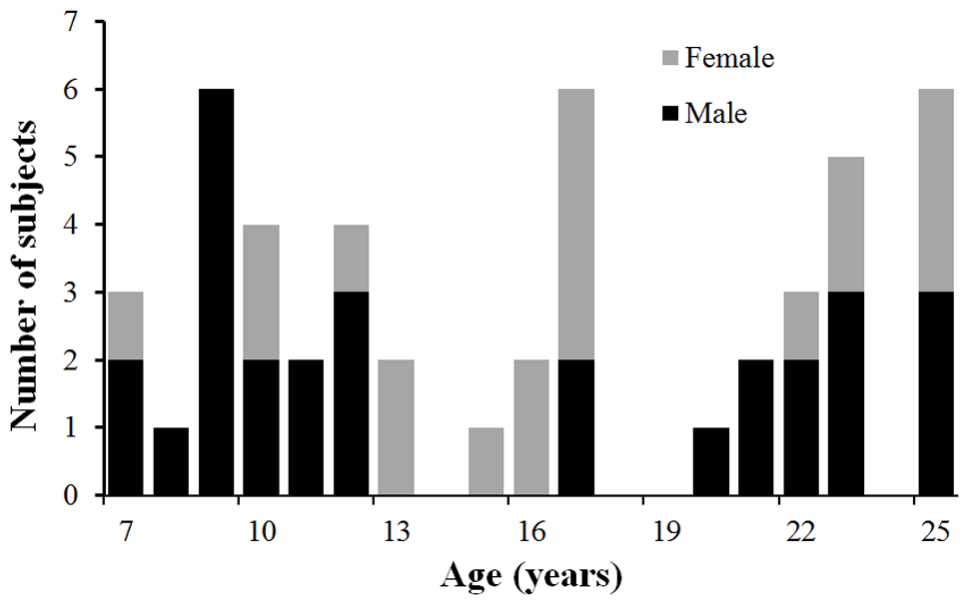
**The age and gender profile of the participated subjects.** Males are in black and females in gray.

A total of 50 healthy children, adolescents and young adults between the ages of 7 and 25 years (31 Male and 19 Female; mean age 16.3 years, standard deviation=6.0 years) were recruited and scanned at the Advanced Imaging Research Center of The University of Texas Southwestern Medical Center (UTSW) at Dallas. **Figure 1** shows the profile of the age and gender of the participated normal subjects. The study was approved by the Institutional Review Board (IRB) at the UTSW. Subjects or their guardians (if subjects are under 18 years old) gave written informed consents for all study procedures. All subjects gave written informed consent. All participants were medically healthy and had no known neurological or psychiatric disorders. They were not under any intervention or medication known to affect the central nervous system. There was no significant correlation between age and gender (*p* = 0.42).

### MRI Data Acquisition

All MRI datasets were obtained on a Philips 3T Achieva MR system (Philips Healthcare, Best, The Netherlands). Diffusion weighted images (DWI) were acquired using a single-shot EPI with SENSE parallel imaging scheme (Sensitivity Encoding reduction factor = 2.3). Diffusion parameters were as follows: FOV = 224/224/143 mm, in plane imaging matrix = 112 × 112, axial slice thickness = 2.2 mm, *TE* = 97 ms, *TR* = 4.41 s, 30 independent diffusion-weighted directions (Jones et al., 1999), with *b*-values of 0 and 1000 sec/mm^2^. To increase signal to noise ratio (SNR), two repetitions were performed, resulting in a total scan time of 11 min for DWI acquisition. T1-weighted magnetization-prepared rapid gradient-echo (MPRAGE) images with FOV = 256/256/160 mm and resolution 1 mm × 1 mm × 1 mm were also acquired. The MPRAGE images provide superior gray and WM contrast and were used for CT measurement and parcellation of the cerebral cortex. DTI and T1-weighted images were acquired in the same session.

### Cortical Parcellation and Thickness Measurement of the Parcellated Cortical Regions

**FIGURE 2:**
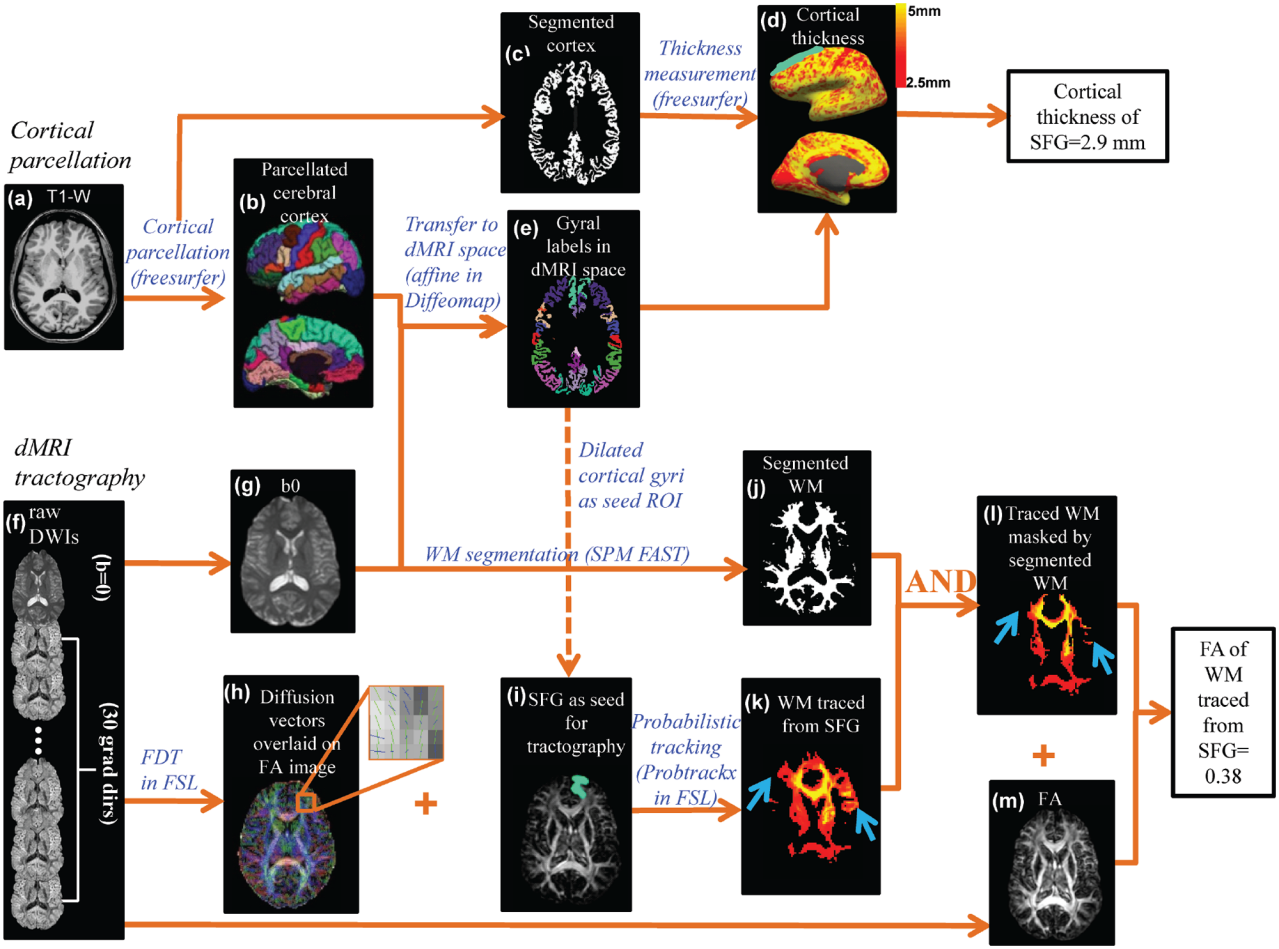
**The data analysis workflow of the cortical parcellation, Cortical thickness (CT) at different gyri and fractional anisotropy (FA) of white matter (WM) fibers traced from the same cortical gyri were measured by integrating information of T1-weighted image **(a)** and diffusion MRI (dMRI) **(f)**.** Based on the T1-weighted image **(a)**, the gray matter was segmented **(c)** and CT **(d)** of a specific cortical gyrus (e.g., superior frontal gyrus or SFG) was measured using the *FreeSurfer* software suite. The cortical surface was parcellated using *FreeSurfer* gyral labeling **(b)** which was transformed to dMRI space using linear affine transformation **(e)** with the b0 of dMRI as the transformation target **(g)**. Probabilistic tracing was implemented using *Probtrackx* in FSL. An enlarged view **(h)** of crossing fiber bundles in the superior frontal region showed crossed fibers could be delineated. A cortical gyral region of interest (ROI) after being dilated by five voxels **(i)** was chosen as a seed for probabilistic fiber tracing **(k)**. WM segmentation **(j)** obtained from FAST segmentation of the b0 image was used to retain only WM and exclude the traced regions entering the gray matter or cerebrospinal fluid **(l)**. The blue arrows in **(k)** and **(l)** point to the region where non-WM voxels were removed from the traced WM. Combined with the FA map **(m)**, FA of traced and filtered WM was then calculated.

The pipeline for measuring thickness of a certain cortical gyrus and WM microstructure traced from the same cortical gyrus is demonstrated in **Figure 2**. Based on T1-weighted image of each subject (**Figure 2a**), the brain cortical surface of each hemisphere was rendered and parcellated into 33 gyral labels (Desikan et al., 2006; **Figure 2b**) using *FreeSurfer* (version 5.0.1)^1^, a semi-automated software suite. After segmentation of brain into gray matter (**Figure 2c**), WM, and cerebrospinal fluid (CSF) using *FreeSurfer*, the distance between the pial surface and GM-WM boundary at a certain cerebral cortex vertex was measured as the thickness of this vertex. The CT map is shown in **Figure 2d**. The thickness values of all cortical vertices within a particular gyral label were then averaged to obtain the thickness of a certain gyrus.

### Quantification of Microstructure of WM Fibers Traced from a Certain Cortical Gyrus

As also shown in **Figure 2**, there are two key components to compute the FA measurement of the WM fibers projected from a cortical gyrus, namely cortical parcellation determining the tractography seed region of interest (ROI) and probabilistic tracking determining the WM traced from a certain cortical gyrus (superior frontal gyrus or SPG was used as an example). For the first component, the parcellated cortical gyrus from the T1-weighted image (**Figure 2b**) was transformed to dMRI space (**Figure 2e**) to serve as the seed ROI for probabilistic tracing (**Figure 2i**). Linear affine transformation was applied to reorient and transform the parcellated cortical labels into dMRI space with the skull-stripped b0 image (**Figure 2g**) and skull-stripped T1-weighted image as the transformation target and subject, respectively, with *Diffeomap*^2^. The same linear transformation re-slices the gyral labeled image using nearest neighbor interpolation. The parcellated cortical ribbon in dMRI space (**Figure 2e**) was then dilated by 5 mm using custom software written in IDL (Interactive Data Language 8.2.3^3^) to penetrate the superficial WM and reach the deep WM to initiate fiber tracking. For the second component, after eddy current correction with FLIRT, raw dMRI (**Figure 2f**) was processed with *BedpostX* of FDT of FSL^4^ to generate the diffusion vectors (**Figure 2h**) and FA (**Figure 2m**) map. With *Probtrackx* in FDT of FSL, probabilistic tracing was conducted with the dilated cortical gyrus as the seed ROI (described above), as shown in **Figures 2i,k**. A threshold of 100 for fdt_paths was applied to retain only WM with high probability of tracing. In addition, a WM mask (**Figure 2j**) segmented with SPM FAST segmentation tool (Statistical Parametric Mapping 8.0^5^, WM threshold =0.8) was generated to filter out spurious tracings reaching to non-WM regions. The filtered WM is shown in **Figure 2l**. Together with the FA map in **Figure 2m**, the FA value of the WM traced from a certain gyrus was calculated after applying a FA threshold of 0.2.

### Evaluation of Seed ROI for WM Tractography

Due to the dense WM zones just beneath the infragranular layers of the cortex impeding tracking (Reveley et al., 2015), we evaluated (1) how much dilation from the segmented cortical gyrus with *FreeSurfer* would be sufficient to go through the dense WM zones; and (2) if tracing directly from the WM immediately beneath the dense WM zones will yield the same tractography results. dMRI data of three representative subjects of ages 8, 16, and 25 years old were used for the evaluation. For the first evaluation approach, three representative segmented cortical gyri, namely precentral gyrus, lateral orbitofrontal gyrus and superior frontal gyrus, obtained from *FreeSurfer* were dilated by 1, 2, 3, 4, and 5 mm into the WM. These dilated regions were used as seed ROIs to initiate WM tracing. For the second evaluation approach, similar to the first approach, cortical gyral regions from *FreeSurfer* were first dilated by 2 and 4 mm into the WM, then cortical gyral regions from *FreeSurfer* dilated by 1 mm were subtracted to bypass dense WM zones (Reveley et al., 2015) impeding tracing. Tractography was conducted with the retained WM regions as seed ROIs. To quantify the differences of WM traced from different seed ROIs, we calculated Dice coefficients among traced WM regions. The Dice coefficient (Dice, 1945) or Dice ratio is defined as the positive agreement between two datasets divided by their average size. For quantification of the differences of traced WM regions, the traced WM was binarized and the overlap of two traced WM regions could be calculated as the ratio of number of WM voxels contained in both binary maps divided by average number of WM voxels.

### Linear Fitting of CT and Age, Corresponding WM FA and Age and the Relationship between CT Change Rate and Corresponding WM FA Change Rate Among Different Frontal Gyri

Linear model was used to fit the CT and corresponding WM FA of each of the 16 gyri in the left and right frontal lobe against age for all subjects with the equations below:

(1) $$CT_{\text{i},\text{j}}\text{=}\alpha_{\text{1},\text{i}}\text{+}\beta_{\text{1},\text{i}}t_{\text{j}}\text{+}\varepsilon_{\text{1},\text{i},\text{j}}$$

(2) $$FA_{\text{i},\text{j}}\text{=}\alpha_{\text{2},\text{i}}\text{+}\beta_{\text{2},\text{i}}t_{\text{j}}\text{+}\varepsilon_{\text{2},\text{i},\text{j}}$$

Where *t*_j_ is the age of the *j*th subject; α_1,i_ and β_1,i_ (or α_2,i_ and β_2,i_) are the unknown intercept and slope for CT (or FA) of the *i*th frontal gyrus, respectively; 𝜀_1,i,j_ and 𝜀_2,i,j_ are the error terms for CT and FA, respectively; *j* is from 1 to 50; *i∈{1,2,…,8}* indexes eight left frontal gyri (namely superior frontal, lateral orbitofrontal, caudal middle frontal, rostral middle frontal, pars opercularis, pars orbitalis, pars triangularis, and precentral gyrus) while *i∈{9,10,…,16}* corresponds to the eight right frontal gyri. The regional analysis was controlled for multiple comparisons using the Benjamini and Hochberg adjustment method (Benjamini and Hochberg, 1995).

Estimates of β_1,i_ and β_2,i_, the unknown rates of CT and FA changes, can be obtained by fitting the regression models in Equation (1) and (2), and the estimators are denoted by 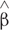
_1,i_ and 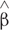
_2,i_, respectively. After standardizing the slopes 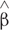
_1,i_ and 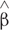
_2,i_, a Deming regression (R package ‘MethComp’) to account for measurement errors in both slopes was used to test if these two rates at all frontal gyri were significantly correlated.

(3) $${\hat{\beta }}_{\text{2},\text{i}}=c+d{\hat{\beta }}_{1,\text{i}}+\;\delta_{\text{i}}$$

Note that 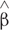
_1,i_ and 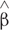
_2,i_ are estimates of the “true” or (expected) values of β_1,i_ and β_2,i_, respectively, at *i*th frontal gyrus. All statistical analysis was computed using R statistical software version 3.0.2^6^.

## Results

### Significant and Heterogeneous Decreases of CT at Different Frontal Gyri during Development

**FIGURE 3:**
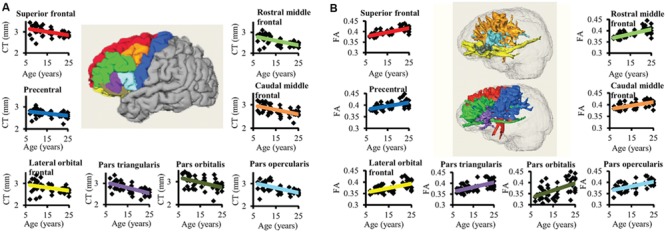
**The age-dependent CT decreases and corresponding WM FA increases for eight left frontal regions are shown in **(A)** and **(B)**, respectively.** In the center of **(A)**, parcellated left frontal gyri were encoded with following colors: superior frontal (red), precentral (blue), lateral orbital frontal (yellow), pars triangularis (purple), pars orbitalis (dark green), pars opercularis (light blue), caudal middle frontal (orange), and rostral middle frontal (green). The colors of the fitted CT decrease trend lines in **(A)** are consistent to those of parcellated gyri in the center. In **(B)**, the three-dimensionally reconstructed WM tracts used for FA measurement in the center were traced from corresponding frontal gyrus and encoded with the same colors as those shown in the parcellated left frontal gyri in the center of **(A)**. The same color scheme used in the fitted trend lines in **(A)** was used for the fitted trend lines in **(B)**.

**FIGURE 4:**
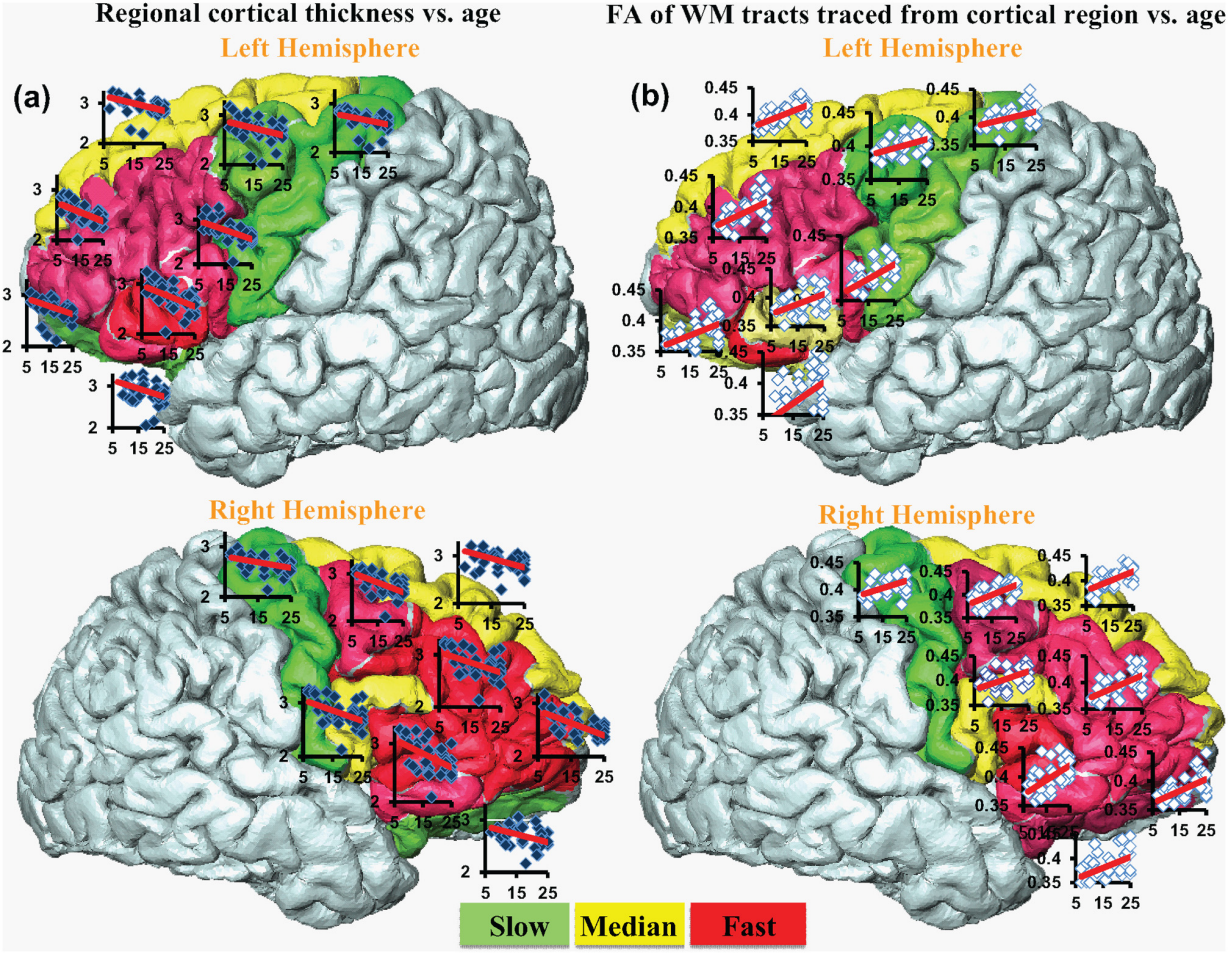
**The coherent CT decrease rate **(a)** and corresponding WM FA increase rate **(b)**.** Each cortical region is encoded by the change rate and categorized into slow (green), median (yellow) and fast (red) from smallest to largest. Scatter plots for regional CT vs. age (blue dots) and corresponding FA vs. age (white dots) are overlaid on the individual gyrus.

Significant age-dependent CT decreases in all eight gyral regions in the left frontal cortex (**Figure 3A** and **Table 1**) and five gyral regions in the right frontal cortex were found (**Table 1**). Besides significant CT decreases, regional heterogeneity of the CT temporal courses, specifically dramatically different CT decrease slopes, can also be observed in **Figure 3A** and **Table 1**. The heterogeneity of the CT decrease in the frontal lobe was indicated by the colors painted on each frontal lobe gyrus in **Figure 4a**. Specifically, the green, yellow, and red colors represent slow, median and fast age-dependent CT decrease, respectively (**Figure 4a** and **Table 1**). For example, in the left frontal lobe, the CT decreases faster in the left pars triangularis and left rostral middle frontal regions, indicated by red, while the CT decreases slower in the left precentral and lateral orbitofrontal gyri, indicated by green (**Figure 4a** and **Table 1**). The CT decrease slopes, correlation coefficients and *p*-values in all frontal gyri are listed in middle column of **Table 1**. The categorization of slow, median and fast CT decreases of all frontal gyri can be found in the CT decrease rate histogram (upper panel of Supplementary Figure S1).

**Table 1:** Pearson correlational coefficient (*r*), correlation significance (*p*) and slopes of fitted linear trend line are shown for age-dependent cortical thickness (CT) (middle column) and corresponding white matter (WM) fractional anisotropy (FA; right column) for all frontal lobe gyri in the left (lh) and right (rh) hemisphere.

	CT vs. age			FA vs. age		
Cortical region	*r*	*p*	Slope (mm/yr)	*r*	*p*	Slope (/yr)
lh-superiorfrontal	0.72	**2.3E-09**	-0.0165	0.90	**5.4E-20**	0.00201
lh-rostralmiddlefrontal	0.42	**2.1E-03**	-0.0219	0.38	**6.0E-03**	0.00213
lh-caudalmiddlefrontal	0.37	**7.3E-03**	-0.0192	0.55	**2.4E-05**	0.00153
lh-parsopercularis	0.46	**6.9E-04**	-0.0166	-0.07	0.604	0.00173
lh-parsorbitalis	0.65	**2.5E-07**	-0.0217	0.72	**2.6E-09**	0.00327
lh-parstriangularis	0.70	**7.2E-09**	-0.0260	0.81	**3.8E-13**	0.00217
lh-lateralorbitofrontal	0.40	**3.3E-03**	-0.0119	0.16	0.250	0.00226
lh-precentral	0.45	**8.1E-04**	-0.0090	0.74	**2.6E-10**	0.00153
rh-superiorfrontal	0.59	**5.2E-06**	-0.0104	0.86	**4.7E-16**	0.00194
rh-rostralmiddlefrontal	0.27	0.056	-0.0165	0.40	**3.1E-03**	0.00231
rh-caudalmiddlefrontal	0.16	0.250	-0.0132	0.51	**1.0E-04**	0.00202
rh-parsopercularis	0.41	**2.9E-03**	-0.0168	0.17	0.241	0.00210
rh-parsorbitalis	0.62	**1.1E-06**	-0.0197	0.52	**8.2E-05**	0.00219
rh-parstriangularis	0.61	**1.3E-06**	-0.0181	0.77	**3.5E-11**	0.00265
rh-lateralorbitofrontal	0.05	0.707	-0.0103	-0.12	0.449	0.00194
rh-precentral	0.47	**4.1E-04**	-0.0078	0.78	**7.8E-12**	0.00151

*The underlined bold indicates *p* < 0.05 after FDR correction.*

### Significant and Heterogeneous Increases of FA of WM Traced from Parcellated Cortical Gyri during Development

Significant age-dependent increases of FA were found for WM traced from a majority of parcellated left and right frontal cortical gyri. **Figure 3B** shows the age-dependent increase in FA for WM traced from all eight left frontal gyri. Similar to heterogeneous age-dependent CT changes, heterogeneity in the FA increase rate among different frontal gyri were observed (**Figure 3B** and **Table 1**). FA values of WM traced from certain frontal gyri, such as the left pars orbitalis and right pars triangularis increase faster compared to those traced from other frontal gyri (**Table 1**). **Figure 4b** demonstrates the change rates of FA of WM traced from frontal cortical gyri, using the same slow, median, and fast color scheme as those in the CT change rates. As shown in **Figures 4a,b**, WM FA increase rate and CT decrease rate appear to be coherent, shown by similar color profile all over frontal gyri. For example, the CT of the precentral gyrus decreases slowly, while, coherently, and FA of the WM traced from precentral gyrus increases slowly, with the green color shown on this gyrus in both **Figures 4a,b**. The FA increase slopes, correlation coefficients and p values in all frontal gyri are listed in the right column of **Table 1**. The categorization of slow, median and fast FA increases for WM traced from all frontal gyri can be found in the FA increase rate histogram (lower panel of Supplementary Figure S1).

### Coherent CT Decrease and Corresponding WM FA Increase

**FIGURE 5:**
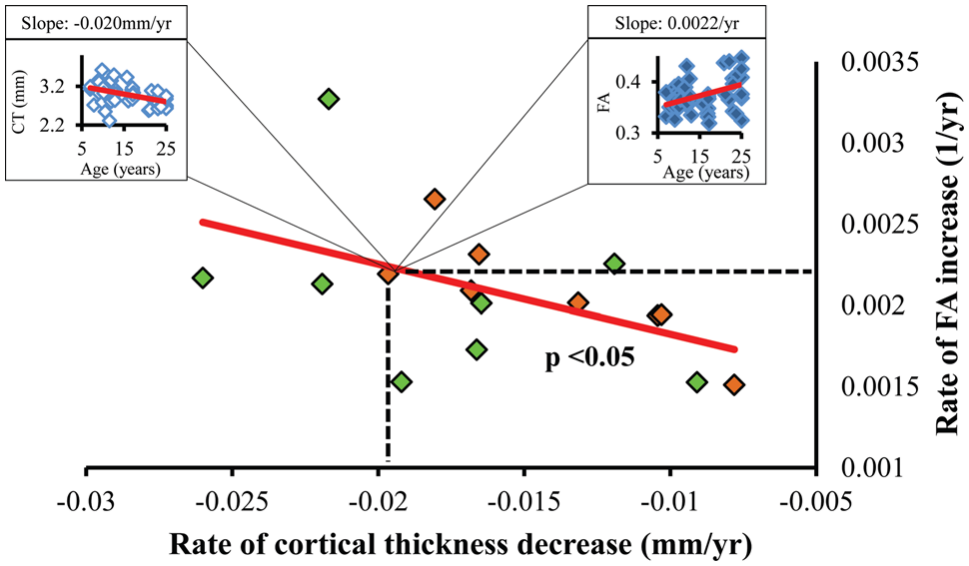
**Statistically significant (*p* < 0.05) linear relationship between the regional CT decrease rate (*x*-axis) and corresponding WM FA increase rate (*y*-axis).** Each dot represents the CT decrease rate and corresponding WM FA increase rate of a left (green dots) or right (orange dots) frontal gyrus. As an example, a dot representing the right pars orbitalis region can be expanded to an age-dependent scatter plot of CT (left box) and that of corresponding WM FA (right box).

Significant correlation between the slopes of CT decrease and those of corresponding WM FA increase were found, as shown in **Figure 5**. Each point in scatter plot of **Figure 5** represents the CT change rate and corresponding WM FA change rate for one frontal gyrus. Despite long physical distances in the WM axons projected from the cortex, including longitudinal, association, and callosal fibers, the correlation of CT change rate and corresponding WM FA change rate is statistically significant (*p* < 0.05), suggesting coherence of cortical structural changes and corresponding WM microstructural changes during frontal lobe development. In **Figure 5**, except the points for left caudal middle frontal gyrus, left pars opercularis and right pars triangularis, all other points lie within the 95% confidence interval. The same analysis pipeline shown in **Figure 2** has been applied to all gyri in the entire brain (data not shown). Significantly coherent changes of WM FA and CT were only found for cortical gyri in the left and right frontal lobe, while no significant coherent changes of WM FA and CT were found for all other cortical lobes.

### Evaluation of the WM Fibers Traced from Varying Seed ROIs

**FIGURE 6:**
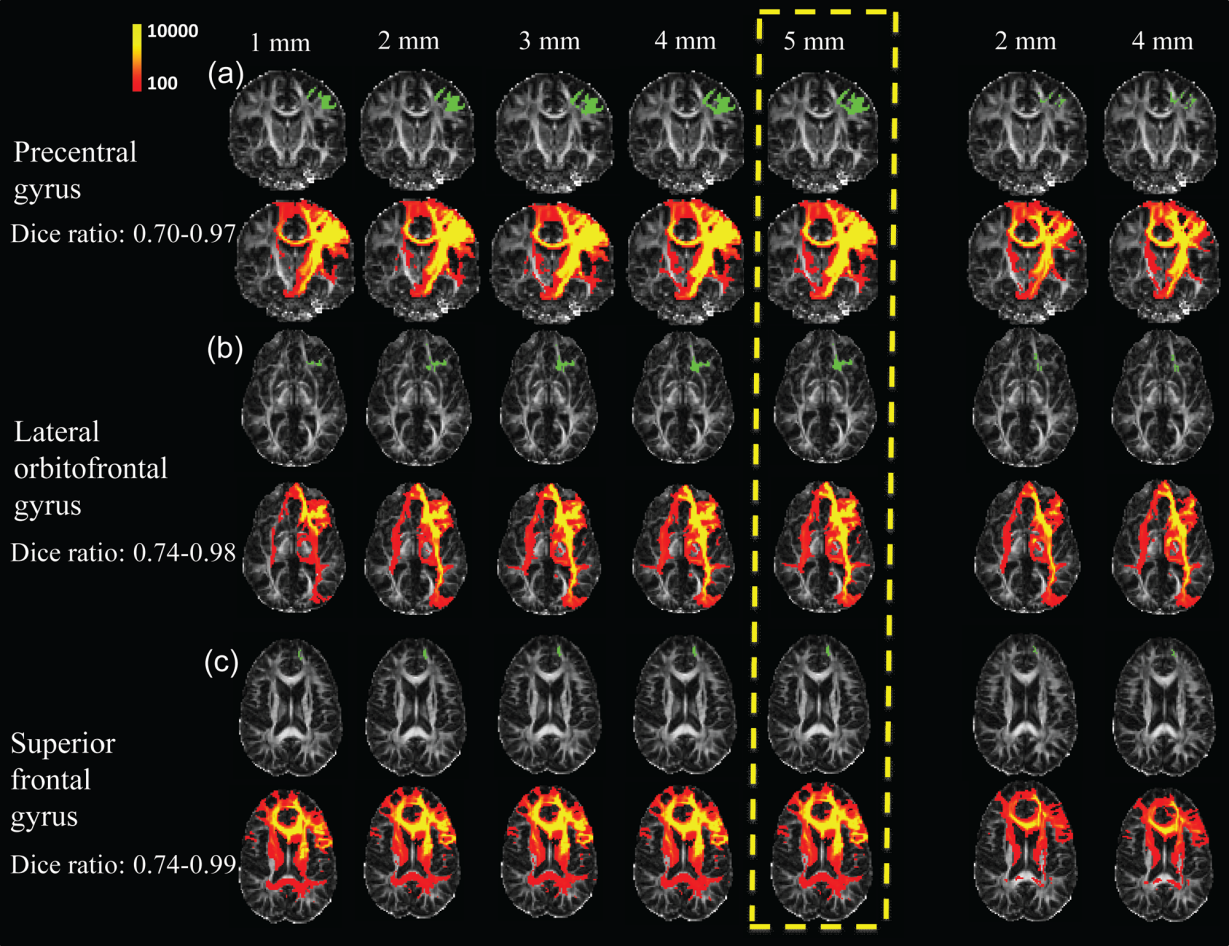
**Evaluation test results of WM fibers traced from three representative cortical gyri namely the precentral gyrus **(a)**, lateral orbitofrontal gyrus **(b)**, and superior frontal gyrus **(c)**, assessed by adjusting the seed ROIs used for WM tracing.** Two approaches were used to adjust seed ROIs. First, the parcellated cortical gyri were dilated, for distances of 1, 2, 3, 4, and 5 mm (left 5 columns), into the WM. For the second approach, the seed ROIs to initialize tracing were inside WM (right two columns), which were obtained by dilating parcellated cortical gyri by 2 and 4 mm into the WM, and subsequently subtracted by the corresponding cortical gyri dilated by 1 mm. The seed ROIs are shown in green with the WM fibers traced from these ROIs shown in red and yellow directly below them. The upper left color bar encodes the tractography probability. The parcellated cortical gyri dilated by 5 mm was used as seed ROI in the present study, highlighted in yellow dashed box.

**Figure 6** shows the results of the WM fibers traced from different seed ROIs for evaluation on how these seed ROIs affect tractography results. **Tables 2A–C** list the Dice ratios of the tractography results from three representative frontal gyri, namely left precentral gyrus (a), left lateral orbitofrontal gyrus (b), and left superior frontal gyrus (c), respectively. **Figure 6** and **Table 2** indicate that distance of dilation from pure cortical regions into WM or tracing directly from WM close to the cortical regions has relatively small effects on tractography results, reflected by similar tractography results in **Figure 6** and similar Dice ratios in **Tables 2A–C**. Changing the seed ROIs led to slight variations in tractography results for these three representative frontal cortical regions, as shown in **Figure 6**. From **Table 2**, mean Dice ratios for all seed ROIs were in the range of 70–99%. Slightly more traced WM fibers were associated with seed ROIs with larger dilation into WM. In the present study, we intended to trace as much WM as possible, therefore, dilated the cortical gyrus by 5 mm into the WM. Furthermore, the pathways and connectivity of all traced WM fibers are consistent to those of known major WM tracts (Mori et al., 2008). For instance, in WM fibers traced from the precentral gyrus, parts of the superior longitudinal fasciculus and callosal fibers, corticospinal tract and short association tracts can be identified.

**Table 2:** Mean Dice ratios and standard deviations of the WM fibers traced from the left precentral gyrus (A), left lateral orbitofrontal gyrus (B), and left superior frontal gyrus (C) using two different evaluation tests by adjusting the seed region of interest (ROI) for WM tracing.

	WM traced from a ROI in the dilated cerebral cortex					WM traced from a ROI inside the WM	
(mm)	1	2	3	4	5	2	4
**A**							
3		0.88 ± 0.10	0.79 ± 0.15	0.78 ± 0.17	0.76 ± 0.15	0.70 ± 0.15	0.73 ± 0.17
5			0.92 ± 0.04	0.90 ± 0.06	0.89 ± 0.04	0.77 ± 0.17	0.84 ± 0.09
7				0.96 ± 0.02	0.94 ± 0.01	0.72 ± 0.20	0.86 ± 0.09
9					0.97 ± 0.02	0.72 ± 0.17	0.85 ± 0.10
11						0.70 ± 0.16	0.84 ± 0.09
2							0.82 ± 0.10
4							
**B**							
3		0.95 ± 0.01	0.92 ± 0.00	0.91 ± 0.01	0.90 ± 0.00	0.80 ± 0.07	0.85 ± 0.06
5			0.97 ± 0.01	0.96 ± 0.00	0.95 ± 0.02	0.78 ± 0.07	0.78 ± 0.06
7				0.98 ± 0.00	0.98 ± 0.01	0.75 ± 0.07	0.84 ± 0.04
9					0.98 ± 0.02	0.75 ± 0.06	0.83 ± 0.04
11						0.74 ± 0.07	0.80 ± 0.06
2							0.90 ± 0.03
4							
**C**							
3		0.96 ± 0.01	0.95 ± 0.01	0.95 ± 0.01	0.95 ± 0.02	0.79 ± 0.05	0.84 ± 0.06
5			0.98 ± 0.00	0.98 ± 0.01	0.98 ± 0.01	0.75 ± 0.05	0.81 ± 0.06
7				0.99 ± 0.00	0.98 ± 0.01	0.74 ± 0.06	0.79 ± 0.07
9					0.99 ± 0.01	0.74 ± 0.06	0.79 ± 0.07
11						0.74 ± 0.07	0.79 ± 0.07
2							0.94 ± 0.01
4							

*In the left five columns, mean Dice ratios and standard deviations are shown for a ROI from the cortex after being dilated by 1, 2, 3, 4, and 5 mm inward into the WM to initialize tracing. In the right two shaded columns, mean Dice ratios and standard deviations from a ROI at the interior WM are listed. The interior WM seed ROI was obtained by dilating a parcellated cortical gyrus by 2 and 4 mm into the WM, and subsequently subtracted by the corresponding cortical gyri dilated by 1 mm. Dice ratio results from three subjects with the representative ages (8, 16, and 25 years of age) spanning the age range in the present study were averaged.*

## Discussion

The present study revealed coherence between the CT decrease and corresponding WM FA increase in the frontal lobe during development, which might suggest synchronous cortical pruning and axonal microstructural enhancement. Both cortical thinning and WM microstructural enhancement are essential age-dependent structural processes during normal development from 7 to 25 years of age. Alterations of either of these processes may be related to neuropathological states. Most human brain WM consists of axons projected from neurons in the cerebral cortex, which is likely to underlie our findings of significant correlation between cortical thinning and corresponding WM microstructural enhancement measured by FA increase. To the best of our knowledge, this study marks one of the first attempts to reveal coherence between changes of CT and those of FA of WM connected to a particular cortical gyrus in normal developing brains.

**FIGURE 7:**
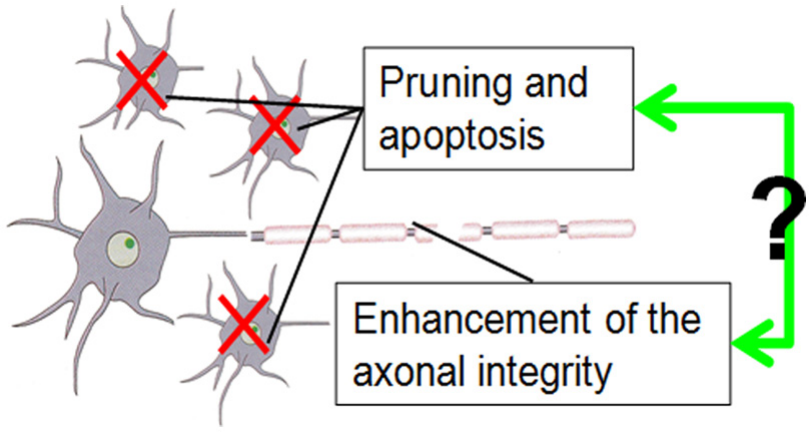
**The cartoonography demonstrates our assumption of possible interaction of apoptosis reflected by CT decrease and enhancement of axonal integrity reflected by FA increase during brain development**.

Both age-dependent changes of CT and WM FA, as structural markers of brain maturation, have been related to functional changes during development from childhood to adulthood. It is then likely that the developmental changes of these two structural markers, CT and WM FA, are correlated. For example, executive function in subjects from 8 to 19 years of age has been associated with CT decreases in the frontal lobe (Tamnes et al., 2010b). CT decreases in the left frontal areas coincide with cognitive ability changes affecting intelligence quotient (IQ) in children and adolescents (Burgaleta et al., 2014). On the other hand, the relationship of WM FA increase and enhanced brain function has also been revealed. For example, it has been shown that enhanced WM microstructure (i.e., increased FA) is associated with better performances on inhibitory control and cognitive flexibility tests for children between the ages of 5 to 16 years old (Treit et al., 2014). Increased FA in major WM tracts is correlated with enhanced neurocognitive performance in healthy subjects between the ages of 8 and 25 years old (Peters et al., 2014). Due to the embryonic link between the cortical interstitial neurons (Von Economo and Koskinas, 2008) and WM axons projected from them, it is intuitive to suggest that age-dependent structural changes of cortex (where the neuronal bodies are located) and those of corresponding WM axons are linked. Primate studies suggest that axon guidance signaling most likely contributes to weak or excessive pruning after the peak has been reached in puberty (Oga et al., 2013; Sasaki et al., 2014). The exact molecular and cellular mechanisms of these coherent changes of CT and WM integrity in development are not known. We speculated that there are some overlapping signaling pathways of axonal guidance and synaptic pruning or neuron apoptosis. A very simplified mechanism is illustrated in the cartoonography in **Figure 7**.

Besides neuroscientific insight of normal brain development, the present study may shed light on the mechanism of abnormal WM development and its association with the cerebral cortex in different neuropsychiatric disorders such as autism and schizophrenia. In post-mortem studies of schizophrenia, a decrease in frontal cortical volume in adolescence caused by a decrease in the number of synapses and reduction in neuropil was found (Selemon et al., 1995). Decreased FA of the uncinate fasciculus, cingulum bundle and arcuate fasciculus (see e.g., Kubicki et al., 2007 for review) was found to be associated with schizophrenia. Abnormalities in both CT and WM microstructure in schizophrenia suggest a link between the developmental processes of these two structural markers (Paus et al., 2008). Similarly, abnormal WM FA and CT were found for autistic brains. Reduced FA was found for WM adjacent to cortical regions implicated in social cognition as well as WM near the prefrontal cortex (Barnea-Goraly et al., 2004). Significant localized CT reductions within the fronto-striatal network was found for the subjects with autism spectrum disorder (Ecker et al., 2014). Revealing coherence of CT and WM microstructural changes during development could open a new window for understanding underlying mechanism of interacted abnormal CT decrease and WM disruption in mental disorders.

The approach to “trace” WM fibers directly from the cortical gyrus in the present study may be a more connectivity-driven one, which differs from those in previous studies focusing on measuring FA in the superficial WM region “adjacent to” certain cortical gyri (Tamnes et al., 2010a; Wu et al., 2014). In the present study, FA values of WM axons traced from a specific cortical region were measured. These traced WM axons could either project from or get to the neurons in this specified cortical region. The present approach is directly linked to connectivity. Despite that traced WM fibers could have long physical distances away from the seed cortical region, significant correlation between CT changes in the frontal regions and FA changes for WM traced from these regions during development was found (**Figures 4** and **5**). On the other hand, the anatomical adjacency in the previous studies does not ensure biological association of the WM axons and the cortical regions. As a result, no significant associations between CT and superficial WM FA were identified, while age-dependent changes of CT and superficial WM FA were found to be significant (Tamnes et al., 2010a; Wu et al., 2014).

Significant correlation between CT and associated WM microstructural changes was not found for cortical regions other than frontal areas (data not shown). Vast regional differences in cell diversity within the cortex in development have been quantified previously by Conel (1941, 1947, 1955, 1959, 1963, 1967). The densities of the interstitial neurons giving rise to most axonal projections are highest (Meyer et al., 1992; Smiley et al., 1998) in the frontal lobe, which may contribute to the observation that coherence was only found in the frontal lobe. Histological findings in primates and humans suggest asynchronous patterns of synaptogenesis (Bianchi et al., 2013) and different size, dendritic branching patterns, spine density, and complexity of pyramidal cells (Hof and Morrison, 1995; Elston, 2000, 2003; Jacobs et al., 2001; Jacobs and Scheibel, 2002) in the prefrontal cortex. This regional variation of pyramidal cells are likely to influence underlying circuitry and functional specializations (Elston et al., 2002; Elston, 2003; Elston and Fujita, 2014). Other related factors may include the accelerated growth of the frontal areas during the studied time period (7–25 years; Giedd et al., 1999; Sowell et al., 2001). In addition, different tracing of superficial WM fibers (Oishi et al., 2008), located in the WM region immediately inside the dense WM zone (details in Reveley et al., 2015), among different lobes through dMRI tractography may also play a role. Although the mechanism is not completely known, heterogeneity in superficial WM fiber directions among different lobes affects dMRI tractography. Despite that probabilistic tractography was used, it is a general limitation that dMRI tractography cannot trace through the areas with mixed fibers in many different directions.

This study may serve as a bridge between age-dependent CT or WM FA changes and age-dependent changes of network metrics based on graph theory. The application of graph theory to study human brain networks termed the “human connectome” (Bullmore and Sporns, 2009) has immensely advanced our understanding of developmental brain circuits. For example, studies on the development of structural (Hagmann et al., 2010; Huang et al., 2015) connections have revealed enhanced network properties including efficiency and network strength. Regional CT and WM FA of developing brains offered insight of regional or local age-dependent structural changes. Metrics based on graph theory provided the global view of age-dependent brain configuration changes. Brain development is a highly complicated yet organized process. The coherent CT and WM FA changes found in this study may be the link between the local age-dependent structural changes and global age-dependent network metric changes.

The current approach of tracing WM initialized from a certain cortical gyrus is extended from our previous study mapping the WM axons initialized from a certain cortical lobe to the cortical surface (Huang et al., 2011). Evaluation tests were performed with computation of the Dice ratios by adjusting the seed ROI for WM tracing. As discussed in the literature (Zhang et al., 2010), it is difficult to determine the accuracy of the traced WM fibers, but the selection of seed ROIs can be optimized to capture most of the WM fibers traced from a cortical gyrus. We compared two different types of cortical seed ROIs to initialize WM fiber tracing. The first type of seed ROIs were directly dilated from relatively “pure” cortex into the WM. For the second type of seed ROI, the cerebral cortex and superficial WM systems directly underlying the boundary between gray and WM were excluded and fiber tracing was initialized from more interior WM. These comprehensive evaluation tests of seed ROIs were conducted due to the dense WM zones directly beneath the infra-granular layer of the cortex that pose a significant barrier to tracing (Reveley et al., 2015). From the test results, only minor differences were found using two different approaches to trace the WM fibers (**Figure 6** and **Table 2**). Depending on different cortical gyri as seed regions, Dice ratios ranged from 70 to 99%. To ensure that traced WM from a cortical ROI accurately represents “true” WM connected to this cortical ROI, the accuracy of tracing WM axons with dMRI may be evaluated by comparison to chemical tracing in the future work.

Methodological limitations are related to the fitting model of age-dependent CT or FA changes, sample size and cross-sectional nature. To reveal the rate of CT or WM FA changes, linear model was used for this selected age-range from mid-childhood to adulthood, where significantly linear changes of CT and FA were observed (**Figure 3** and **Table 1**). Although a quadratic or cubic fitting was used for CT changes over a larger age range, namely 3.5–33 years old (Shaw et al., 2008), linear decrease of CT during the age range of 7–25 years of age in the present study was suggested by the literature (Shaw et al., 2008). Similarly, FA increases followed an exponential or quadratic fit in the age range of 5–32 years (Lebel et al., 2008; Lebel and Beaulieu, 2011). For a narrower age range of 725 years, these same studies suggest linear FA increases. With the age range spanning 18 years, the sample size of 50 in the present study is relatively small. However, it is noteworthy that despite the small sample size, the significant correlation of changes (i.e., slopes) of CT and those of FA for WM traced from corresponding frontal cortical regions could be differentiated from non-significant correlation in cortical regions other than frontal lobe. Cross-sectional data introduced considerable individual variation into the age-dependent CT or FA changes and correlations of these two changes. Future longitudinal studies are warranted to reduce individual variations in the dataset.

## Conclusion

The present study revealed the coherence of CT decrease and corresponding WM FA increases during brain development. This finding suggested a possible link of signaling pathways of axonal guidance and synaptic pruning or apoptosis. Revealing synchronous cortical and WM structural changes during development may shed light on understanding underlying mechanisms of normal development and those associated with abnormal CT changes and WM disruption in neuropsychiatric disorders.

## Author Contributions

TJ conducted data analysis and prepared manuscript; VM conducted data analysis; MO conducted data analysis; MC conducted data analysis; HH designed the project, organized data acquisition and prepared manuscript.

## Conflict of Interest Statement

The authors declare that the research was conducted in the absence of any commercial or financial relationships that could be construed as a potential conflict of interest.

## Footnotes

1 http://surfer.nmr.mgh.harvard.edu

2 http://www.mristudio.org

3 http://www.exelisvis.com

4 http://www.fmrib.ox.ac.uk/fsl

5 http://www.fil.ion.ucl.ac.uk/spm

6 http://www.r-project.org
