# Supplementary material for: Synchronous Changes of Cortical Thickness and Corresponding White Matter Microstructure During Brain Development Accessed by Diffusion MRI Tractography from Parcellated Cortex
Source: Front Neuroanat. 2015 Dec 2;9:158. doi: 10.3389/fnana.2015.00158 (PMC4667005; doi:10.3389/fnana.2015.00158)
Supplement: Supplementary file 1 [file Data_Sheet_1.DOCX]

***Supplementary Material***

**Coherent changes of cortical thickness and corresponding white matter microstructure during brain development accessed by diffusion MRI tractography from parcellated cortex**

**Tina Jeon^1,2^, Virendra Mishra^2,3^, Minhui Ouyang^1,2^, Min Chen^4^, Hao Huang^1,5^**

^1^Radiology Research, Children’s Hospital of Philadelphia, Philadelphia, PA, USA

^2^Advanced Imaging Research Center, University of Texas Southwestern Medical Center at Dallas, Dallas, TX USA

^3^Lou Ruvo Center for Brain Health, Cleveland Clinic, Las Vegas, NV, USA

^4^Department of Mathematical Sciences, University of Texas at Dallas, Richardson, TX USA

^5^Department of Radiology, Perelman School of Medicine, University of Pennsylvania, Philadelphia, PA, USA

***Correspondence:** Hao Huang, Ph.D., Radiology Research, Children’s Hospital of Philadelphia, 3401 Civic Center Blvd, Philadelphia, PA, 19104, USA.

huangh6@email.chop.edu

Tina Jeon, Radiology Research, Children’s Hospital of Philadelphia, 3401 Civic Center Blvd, Philadelphia, PA, 19104, USA.

jeont@email.chop.edu

**Supplementary Figure 1:** The histogram of the change slopes of cortical thickness (top panel) and corresponding white matter FA (bottom panel) for each of the 16 frontal lobe gyral regions during development from 7 to 25 years of age. The regions were grouped into 3 clusters, slow (green), median (yellow), and fast (red) based on slope values.
