# Supplementary figures and images for: Synchronous Changes of Cortical Thickness and Corresponding White Matter Microstructure During Brain Development Accessed by Diffusion MRI Tractography from Parcellated Cortex
Source: Front Neuroanat. 2015 Dec 2;9:158. doi: 10.3389/fnana.2015.00158 (PMC4667005; doi:10.3389/fnana.2015.00158)

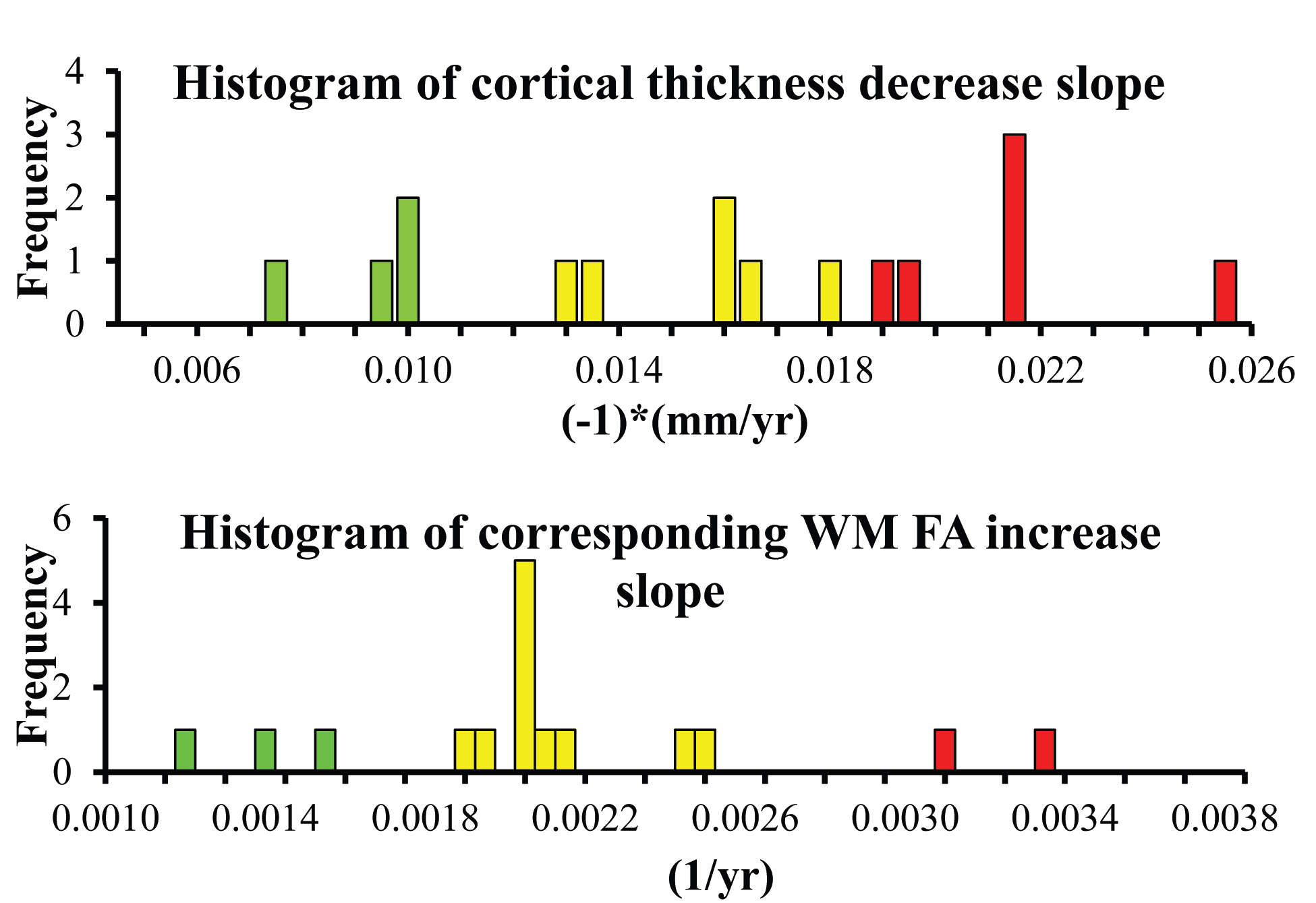

Supplement: Supplementary file 2 [file Image_1.TIF]
